# Internalisation enhances photo-induced cytotoxicity of monoclonal antibody-phthalocyanine conjugates

**DOI:** 10.1054/bjoc.2001.2170

**Published:** 2001-11

**Authors:** M Carcenac, M Dorvillius, V Garambois, F Glaussel, Ch Larroque, R Langlois, N E Hynes, J E van Lier, A Pèlegrin

**Affiliations:** EA2989 Centre de Recherche en Cancérologie, CRLC Val d'Aurelle-Paul Lamarque, Montpellier, F-34298 cedex 5, France; INSERM U128, BP5051, Montpellier, F-34033, France; University of Sherbrooke, Québec, J1H 5N4, Canada; Friedrich Miescher Institute, PO Box 2543, Basel, CH-4002, Switzerland

**Keywords:** immunophototherapy, phthalocyanine, monoclonal antibody, CEA, ErbB2

## Abstract

Immunophototherapy of cancer combines the specificity of a monoclonal antibody (MAb) to an overexpressed tumor marker with the phototoxic properties of the conjugated dye. To analyze the potential role of internalisation of the dye on photo-induced cytotoxicity, we compared two target antigens, carcinoembryonic antigen (CEA) that does not internalise and ErbB2 that does. Human ovarian carcinoma SKOv3 cells that express a high level of ErbB2 were transfected with the CEA cDNA. Using FACS analysis, the resulting cell line, SKOv3-CEA-1B9, demonstrated comparable levels of expression of the two target antigens. Aluminium tetrasulfophthalocyanine (AlPcS _4_) was covalently coupled to anti-CEA MAb 35A7, anti-ErbB2 MAb FSP77 and a non-specific MAb PX, via a five-carbon sulfonamide spacer chain (A _1_) at molar ratios ranging from 6 to 9 moles of AlPcS _4_ per mole of MAb. The 35A7-(AlPcS _4_ A _1_)_8_ conjugate induced 68% growth inhibition of the SKOv3-CEA-1B9 cell line after a 20 h incubation at 2.50 μg/ml (based on AlPcS _4_ A _1_ content) following light exposure. However, the FSP77-(AlPcS _4_ A _1_)_6_ conjugate gave a 51% growth inhibition for an AlPcS _4_ A _1_ concentration as low as 0.04 μg/ml after the same incubation time and exposure to the same light dose. At a 1.25 μg/ml AlPcS _4_ A _1_ concentration, the FSP77-(AlPcS _4_ A _1_)_6_ conjugate gave a 67% growth inhibition after an incubation time as short as 1 h, reaching a 96% inhibition after an 8 h incubation time. Using an unique cell line that expresses two different target antigens, we demonstrated a clear advantage of an internalising over a non-internalising MAb-dye conjugate in terms of phototoxic efficacy. In vivo evaluation of the photodynamic properties of the conjugates is in progress. © 2001 Cancer Research Campaign http://www.bjcancer.com © 2001 Cancer Research Campaign


*British Journal of Cancer* (2001) **85**(11), 1787–1793
© 2001 Cancer Research Campaign
doi: 10.1054/ bjoc.2001.2170, available online at http://www.idealibrary.com on IDEAL

http://www.bjcancer.com

### Internalisation enhances photo-induced cytotoxicity of monoclonal antibody-phthalocyanine conjugates

**M Carcenac[1], M Dorvillius[1], V Garambois[1], F Glaussel[1], Ch Larroque[2], R Langlois[3], NE Hynes[4], JE van Lier[3] and A Pèlegrin[1*]**

[1]EA2989 Centre de Recherche en Cancérologie, CRLC Val d'Aurelle-Paul Lamarque, F-34298 cedex 5 Montpellier, France; [2]INSERM U128, BP5051, F-34033 Montpellier, France; [3]University of Sherbrooke, Québec, J1H 5N4 Canada; [4]Friedrich Miescher Institute, PO Box 2543, CH-4002 Basel, Switzerland

**Summary** Immunophototherapy of cancer combines the specificity of a monoclonal antibody (MAb) to an overexpressed tumor marker with the phototoxic properties of the conjugated dye. To analyze the potential role of internalisation of the dye on photo-induced cytotoxicity, we compared two target antigens, carcinoembryonic antigen (CEA) that does not internalise and ErbB2 that does. Human ovarian carcinoma SKOv3 cells that express a high level of ErbB2 were transfected with the CEA cDNA. Using FACS analysis, the resulting cell line, SKOv3-CEA-1B9, demonstrated comparable levels of expression of the two target antigens. Aluminium tetrasulfophthalocyanine ($AlPcS_4$) was covalently coupled to anti-CEA MAb 35A7, anti-ErbB2 MAb FSP77 and a non-specific MAb PX, via a five-carbon sulfonamide spacer chain ($A_1$) at molar ratios ranging from 6 to 9 moles of $AlPcS_4$ per mole of MAb. The 35A7-$(AlPcS_4A_1)_8$ conjugate induced 68% growth inhibition of the SKOv3-CEA-1B9 cell line after a 20 h incubation at 2.50 µg/ml (based on $AlPcS_4A_1$ content) following light exposure. However, the FSP77-$(AlPcS_4A_1)_6$ conjugate gave a 51% growth inhibition for an $AlPcS_4A_1$ concentration as low as 0.04 µg/ml after the same incubation time and exposure to the same light dose. At a 1.25 µg/ml $AlPcS_4A_1$ concentration, the FSP77-$(AlPcS_4A_1)_6$ conjugate gave a 67% growth inhibition after an incubation time as short as 1 h, reaching a 96% inhibition after an 8 h incubation time. Using an unique cell line that expresses two different target antigens, we demonstrated a clear advantage of an internalising over a non-internalising MAb-dye conjugate in terms of phototoxic efficacy. In vivo evaluation of the photodynamic properties of the conjugates is in progress. © 2001 Cancer Research Campaign http://www.bjcancer.com

**Keywords**: immunophototherapy; phthalocyanine; monoclonal antibody; CEA; ErbB2

**Abbreviations**: MAb, monoclonal antibody; PBS, phosphate-buffered saline; EDC, 1-(3-dimethylaminopropyl)-3-ethyl-carbodiimide hydrochloride; sulfo-NHS, N-hydroxysulfosuccinimide; AlPcS4, aluminium tetrasulfophthalocyanine; CEA, carcinoembryonic antigen; Al, five carbon spacer chain; Px, IgG1 purified from mouse myeloma P3-X63; %ID/g, percentage of injected dose per gram; PS, photosensitizer; IPT, immunophototherapy; mTHPC, metatetrahydroxyphenylchlorin.

Photodynamic therapy (PDT) is a promising new approach for the treatment of superficially localised tumours. This modality involves local or systemic delivery of a photosensitizer (PS), followed by tissue illumination with light of appropriate wavelength. This light causes activation of PS which produces activated oxygen molecules (singlet oxygen), responsible for tumour necrosis. This tumour necrosis is the result of direct and indirect (occlusion of tumour blood vessels, inflammatory reaction) damage to the tumours cells (Dougherty et al, 1998). One of the limitations of this approach is the low selectivity of the PS for tumour tissues. An attractive option to overcome this problem is to couple PS on monoclonal antibodies (MAb) directed against tumour-associated antigens (Goff et al, 1994; Mew et al, 1983; Oseroff et al, 1986; Vrouenraets et al, 1999). In a previous study, we described newly developed photoimmunoconjugates synthesized with an anti-CEA MAb and an hydrophylic PS, aluminium tetrasulfophthalocyanine, $AlPcS_4A_1$ (Carcenac et al, 1999). These conjugates were phototoxic in vitro on CEA-expressing cells, but this photo-induced cytotoxicity was not sufficient to induce tumour necrosis in vivo. We hypothesised that this photo-induced cytotoxicity could be enhance by using a MAb able to deliver the PS intracellularly.

In the present study, we coupled the same PS to an internalising antibody, FSP77, directed against ErbB2. We compared its photo-induced cytotoxicity to that of a non-internalising conjugate directed against CEA on an original cell line, SKOv3-CEA-1B9, expressing the two target antigens, ErbB2 and CEA. Our results confirmed the hypothesis since the anti-Erb2 conjugate was about 10 times more efficient than the anti-CEA conjugate for killing the target cells.

*Received 21 March 2001*
*Revised 2 October 2001*
*Accepted 4 October 2001*

*Correspondence to:* A Pèlegrin

### MATERIAL AND METHODS

### Photosensitizer

Tetrasulfonated aluminium phthalocyanine ($AlPcS_4$) was converted to the tetrasulfonyl chloride derivative and reacted with 6-amino-hexanoic acid to yield the monosulfonamide derivative $AlPcS_4A_1$, bearing a free carboxyl group. After activation and chromatographic purification the material was lyophilized and stored at –20˚C prior to use (Brasseur et al, 1999; Carcenac et al, 1999).

### Monoclonal antibodies

Three murine monoclonal antibodies were used for conjugation with the activated $AlPcS_4A_1$: the anti-CEA MAb 35A7, which was used for immunophotodetection (IPD) (Gutowski et al, 2001; Pèlegrin et al, 1991) and immunophototherapy (IPT) studies (Carcenac et al, 1999); MAb FSP77, which bind specifically to the extracellular domain of the human ErbB2 receptor (Harwerth et al, 1992); and MAb Px purified from mouse myeloma P3-X63 (Kohler et al, 1976), which was used as reference $IgG_1$ for the synthesis of a non-specific immunoconjugate. All the MAbs were purified from mouse ascites using ammonium sulfate precipitation and ion exchange chromatography.

### Preparation of MAb-$AlPcS_4A_1$ conjugates

MAbs were coupled to $AlPcS_4A_1$ by the carbodiimide method as described (Brasseur et al, 1999; Carcenac et al, 1999). Briefly, the monocarboxylic group of $AlPcS_4A_1$ was first activated with 1-(3-dimethylaminopropyl)-3-ethyl-carbodiimide hydrochloride (EDC, Aldrich) and N-hydroxysulfosuccinimide (sulfo-NHS, Pierce). The activated photosensitizer was added drop-wise to a MAb solution (1 mg/ml) in 0.5 M sodium bicarbonate buffer, pH 9.0. The mixture was kept for 18 h at 4˚C in the dark to allow complete coupling of the activated $AlPcS_4A_1$ via formation of amide bonds with the free amino groups of the MAb. The conjugate was separated from excess dye by gel filtration chromatography over a Sephadex G25 column (Pharmacia, Uppsala, Sweden) equilibrated in phosphate-buffered saline (PBS, pH 7.4) containing 0.1% azide. The molar $AlPcS_4$MAb ratios were estimated by spectroscopy using the following equations: $C(MAb) = [A_{280\,nm} - (0.5 \times A_{340\,nm})] / \varepsilon_{MAb}$ and $C(PS) = A_{340\,nm} / \varepsilon_{PS}$ with $\varepsilon_{MAb} = 195{,}000\ M^{-1}\ cm^{-1}$ and $\varepsilon_{PS} = 52{,}400\ M^{-1}\ cm^{-1}$, respectively. This method allowed to calculate an approximate number of dye molecules per Mab molecule. Integrity of the MAb-$AlPcS_4A_1$ conjugates was analyzed by electrophoresis (7.5% SDS-PAGE under nonreducing conditions) in comparison with the native unconjugated MAb.

MAbs and conjugates were radioiodinated using the Iodogen method (1,3,4,6-tetrachloro-3α, 6α-diphenylglycoluryl, Sigma), to arrive at a final specific activity of about 2 µCi of $^{125}I$ per µg protein (1 µCi = 37 GBq) as described (Carcenac et al, 1999). Immunoreactivity of the different $^{125}I$-labeled conjugates was compared to that of the corresponding $^{125}I$-labeled MAbs in a direct binding assay using the relevant antigen coupled to Sepharose-CNBR (Pharmacia) (Carcenac et al, 1999).

### Cell line and transfection

The human ovarian carcinoma cell line, SKOv3, purchased from the American Type Culture Collection (HTB-77, Rockville, MD, USA), was maintained according to recommended procedures. The SKOv3 cells were transfected with pRc/CMV/CEA-cDNA (Pèlegrin et al, 1992) using a mammalian transfection kit (Stratagene, La Jolla, CA, USA). Three µg of DNA were precipitated with calcium phosphate according to the manufacturer's instructions and incubated for 16 h with about $3 \times 10^6$ non-confluent adherent carcinoma cells. Fresh culture medium was then added and after a further 24 h incubation, the cells were harvested, divided in two 75 $cm^2$ flasks and grown for 24 h before adding the Geneticin analogue G418 (Gibco, Paisley, UK) at a concentration of 500 µg/ml. This concentration of G418 was shown to be 5 times the minimal lethal dose for non-transfected SKOv3 cells. Cells resistant to G418 were expanded and tested for CEA expression by flow cytometry using the fluorescence-activated cell sorter (FACS, Becton Dickinson, Mountain View, CA, USA). CEA-positive cells were sorted and cloned by limiting dilution. Among the different CEA-expressing clones obtained, clone 1B9 was named SKOv3-CEA-1B9 and used for further experiments.

### Internalisation of MAb-$(AlPcS_4A_1)_n$ conjugates

Cells SKOv3-CEA-1B9 monolayers were obtained by seeding 3–5 $\times 10^4$ cells in 3 ml of DMEM containing 10% foetal calf serum and supplemented with L-glutamine, on 22-mm square glass coverslips deposited in 35-mm Petri dishes. Three days after culture initiation, cells were washed with PBS, and incubated at 4˚C or 37˚C during 30, 90 and 240 min with the different conjugates FSP77-$(AlPcS_4A_1)_6$, 35A7-$(AlPcS_4A_1)_8$ and Px-$(AlPcS_4A_1)_9$ at 10 µg/ml of Mab. The cells were washed with PBS, and fixed for 20 to 30 min in 3.7% *p*-formaldehyde in PBS, washed with PBS, and permeabilized with acetone at –20˚C for 30 s. After washes with PBS, the cells were incubated with fluorescein isothiocyanate (FITC)-conjugated anti-mouse Ig $F(ab')_2$ fragment (Selenus, AMRAD) during 1 h in the dark. Nuclei were counterstained using DAPI (4,6 diamidino 2–2 phenylindoldihydrochloride, Sigma). The coverslips were deposited on microscope slides with VECTASHIELD® Mounting Medium for fluorescence (VECTOR Laboratories Inc, Burlingame, CA USA). Mounted slides were stored at 4˚C protected from light until use. Slides were observed with an epiillumination fluorescence microscope (Axioplan 2 imaging HBO 100; Zeiss) equiped with a coupled charge device camera (IAI, Progressive Scan, Japan). Images were obtained at × 63, using excitation light provided by a mercury vapor lamp. Analysis of images was performed using appropriate software (Meta systems isis 4, in situ imaging system).

### In vitro photo-induced cytotoxicity

The cytotoxic activity of the immunoconjugates FSP77-$(AlPcS_4A_1)_6$, 35A7-$(AlPcS_4A_1)_8$ and Px-$(AlPcS_4A_1)_9$ was assessed in the SKOv3-CEA-1B9 cell line. After trypsinization, the cells were resuspended in phenol red-free RPMI and dispensed in 96-well plates (20 000 cells/well) and grown for 24 h to allow cells to attach. Two types of experiments were realized. In the first series, after removing the growth medium, FSP77-$(AlPcS_4A_1)_6$, 35A7-$(AlPcS_4A_1)_8$ or Px-$(AlPcS_4A_1)_9$ diluted in phenol red-free RPMI, were added to the cells in triplicates to the final concentrations indicated in Figure 3. After a 20 h incubation, unbound immunoconjugates were removed by washing twice with medium. Fresh phenol red-free RPMI was added, and cells were illuminated at 676 nm with a light source consisting of a focalized Xenon Cermax LX-300F lamp (ILC Technology, Sunnyvale, CA, USA) at a dose of 50 $J/cm^2$. During illumination, the cells were maintained at 37˚C using a Multi-block® Heater (Lab-Line Instruments, Inc). In the second series, after removing the growth medium, FSP77–$(AlPcS_4A_1)_6$, 35A7–$(AlPcS_4A_1)_8$ or Px-$(AlPcS_4A_1)_9$ prepared in phenol red-free RPMI, were added to the cells in triplicates to a concentration of 1.25 µg/ml. After different incubation times (1, 2, 4 and 8 h), unbound immunoconjugates were removed by washing twice with medium. Fresh phenol red-free RPMI was added and cells were illuminated in the same conditions as in experiment one.

 *© 2001 Cancer Research Campaign*

Two days after photoirradiation, the viability of the cells was analysed using the tetrazolium salt 3-[(4,5-dimethylthiazol-2-yl)-2,5-diphenyltetrazolium bromide]) (Sigma) MTT colorimetric assay (Merlin et al, 1992). Briefly, 50 µl of a 0.5% MTT solution was added to each well and incubated for 4 h at 37˚C to allow MTT metabolization. The crystals formed were dissolved by adding 100 µl per well of isopropylic alcohol, HCl 1N. The absorbance at 540 nm was measured on a Microtiter® Plate Reader MRX (Dynatech Laboratories). The results were expressed with respect to control values (cells without any treatment). For all experiments, all the conjugates were not toxic without illumination.

### RESULTS

### In vitro characterization of MAb-AlPcS$_4$A$_1$ conjugates

Conjugates substituted with 6 to 9 moles of AlPcS$_4$ per mole of MAb were obtained by using an initial molar ratio of 40 in the reaction mixture. The percentage of aggregates determined by gel filtration on a Sephacryl 300 column (Pharmacia) was found to be < 10%. The immunoreactivity of all the conjugates determined in a direct binding assay was shown to be similar to that observed with the corresponding unconjugated MAb. SDS-PAGE analysis of the conjugates demonstrated a single protein band with an apparent molecular weight comparable to that of native unconjugated MAb (data not shown).

### Characterization of the SKOv3-CEA-1B9 cell lline

Transfection of the full-length CEA-cDNA into the ErbB2 expressing SKOv3 cell line gave around 15% CEA expressing cells after selection with the neomycin analogue G418. The CEA expressing cells were sorted by FACS and cloned by limiting dilutions. For five clones, CEA expression was comparable to ErbB2 expression. Clone 1B9 was used for the following experiments. Expression of CEA and ErbB2 determined by FACS is shown in

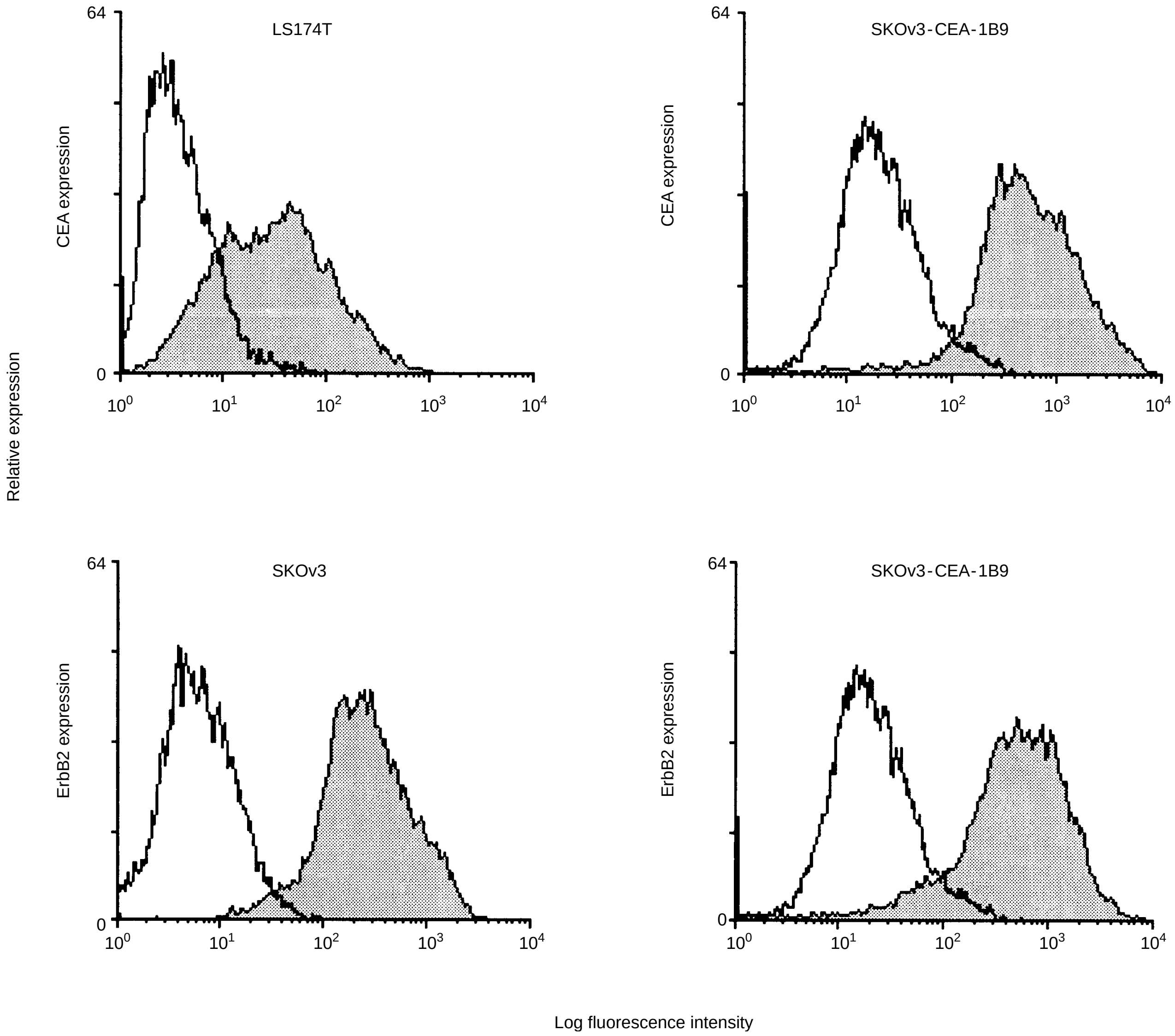

**Figure 1** Flow cytometry analysis of the newly developed SKOv3-CEA-1B9 cell line, and control cell lines LS174T and SKOv3, using anti-CEA MAb or anti-ErbB2 MAb. LS174T is a human colon carcinoma cell line selected for high CEA expression and SKOv3, human ovarian carcinoma was used for transfection. Irrelevant control fluoresceinated IgG (——; no shading), fluoresceinated anti-CEA MAb or anti-ErbB2 MAb (——; black shading)

© 2001 Cancer Research Campaign

Figure 1 for SKOv3-CEA-1B9 as compared with the parental SKOv3 cell line (high ErbB-2 expression) and a reference human colon carcinoma cell line LS174T (high CEA expression). The histograms obtained with the SKOv3-CEA-1B9 cells gave a sharper peak of CEA expression than that obtained with the LS174T cell line, suggesting that the transfected cells gave a more homogeneous CEA expression because they were stably transfected with human CEA cDNA. In the SKOv3-CEA-1B9 cell line, ErbB2 and CEA antigenic densities were very close with fluorescence intensity medians of 500.29 +/– 24.66 and 495.81 +/– 26.73 for ErbB2 and CEA, respectively (Figure 1).

### Internalisation of MAb-$(AlPcS_4A_1)_n$ conjugates

Analysis of the internalisation of the different conjugates was performed using an immunofluorescence technique on the SKOv3-CEA-1B9 cells at 4˚C and 37˚C. Our results confirmed the internalising properties of the anti-ErbB2 conjugate, FSP77-$(AlPcS_4A_1)_6$ as well as the non-internalisation of the anti-CEA conjugate, 35A7-$(AlPcS_4A_1)_8$. At 37˚C, binding of the two conjugates on SKOv3-CEA-1B9 cells was shown after a 30 min incubation time (Figure 2A and D). The intracellular localisation of the FSP77-$(AlPcS_4A_1)_6$ conjugate was seen after the 90 min incubation time (Figure 2B) and intracellular vesicles containing the conjugate were obvious after 240 min (Figure 2C). During this kinetic analysis, the 35A7-$(AlPcS_4A_1)_8$ conjugate did not show any intracellular localisation (Fig 2D–F). At 4˚C, no internalisation was visualised with the FSP77-$(AlPcS_4A_1)_6$ conjugate which gave a membrane binding comparable to that obtained with 35A7-$(AlPcS_4A_1)_8$. No cellular binding was observed with the irrelevant conjugate, Px-$(AlPcS_4A_1)_9$.

### In vitro photo-induced cytotoxicity

The in vitro cytotoxicity of immunoconjugates was evaluated on SKOv3-CEA-1B9, using an illumination at a light dose of 50 J/cm$^2$. In a first series of experiments, cells were incubated 20 h with five different concentrations of photosensitizer ranging from 0.0025 µg/ml to 2.50 µg/ml (Figure 3). The FSP77-$(AlPcS_4A_1)_6$ induced 96% growth inhibition of the cells for the $AlPcS_4A_1$ concentration of 2.50 µg/ml. At the same photosensitizer concentration, 35A7-$(AlPcS_4A_1)_8$ generated a 68% growth inhibition. However, the FSP77-$(AlPcS_4A_1)_6$ conjugate gave a 51% growth inhibition for an $AlPcS_4A_1$ concentration as low as 0.04 µg/ml after the same incubation time and exposure to the same light dose (Figure 3). Experiments realised with native (unconjugated) Mab FSP77 or free $AlPcS_4A_1$, with or without light illumination did not give any cell growth inhibition.

In a second series of experiments, SKOv3-CEA-1B9 cells were incubated for 1, 2, 4 or 8 h with a constant concentration of $AIPcS_4A_1$ of 1.25 µg/ml (Figure 4). The FSP77-$(AlPcS_4A_1)_6$ conjugate gave 67%, 76%, 86% and 96% growth inhibition after 1, 2, 4 and 8 h of incubation, respectively. No photo-induced cytotoxicity was observed with the 35A7-$(AlPcS_4A_1)_8$ conjugate up to 4 h of incubation. With a 8 h incubation time, the 35A7-$(AlPcS_4A_1)_8$ conjugate gave a 72% growth inhibition.

### DISCUSSION

Immunophototherapy is an attractive modality to enhance photodynamic therapy efficacy by increasing the tumor-to-normal tissues ratios of the photosensitisers. In a previous report, we described the preparation and characterisation of MAb-phthalocyanine conjugates directed against CEA (Carcenac et al, 1999). Despite a phototoxic activity in vitro on LoVo cells (91% growth inhibition at 2.5 µg/ml $AlPcS_4A_1$ concentration) and a high tumour uptake (up to 30% ID/g tumour 24 h post-injection), the anti-CEA 35A7-$AlPcS_4A_1$ conjugates had a limited in vivo anti-tumour activity. For this reason, we decided to evaluate MAb-dye conjugates able to deliver photosensitisers inside the cells. Internalisation of such conjugates can be obtained by (i) a chemical modification of the conjugate or (ii) the internalising character of target antigen. We demonstrate here that monoclonal antibody–phthalocyanine conjugates directed against an internalising antigen, ErbB2, are more phototoxic than conjugates directed against a non-internalising antigen, CEA.

Since 1996, the group of Hasan has explored the first possibility using poly-L-lysine linkers to prepare cationic chlorin$_{e6}$ ($c_{e6}$)-antibodies conjugates and succinylated poly-L-lysine linkers to obtain anionic conjugates (Del Governatore, 2000a, 2000b; Duska et al, 1997; Hamblin et al, 1996,2000). In the first part of their work, these authors demonstrated that a cationic conjugate prepared with $c_{e6}$ and F(ab')$_2$ fragments from MAb OC125 induced in vitro a higher photo-induced cytotoxicity on the human ovarian cancer cell line NIH-OVCAR-5 than an anionic conjugate (Hamblin et al, 1996). The positive charges of the cationic conjugate allowed a higher cellular uptake being responsible for the enhanced photo-induced cytotoxicity. When the NIH-OVCAR-5 cells were used to induce a peritoneal carcinomatosis in nude mice, the cationic conjugate was shown to deliver about 2.6 times more $c_{e6}$ in tumor nodules 24 h post i.p. injection than the anionic conjugate as determined by fluorescence ($28.3 \pm 9.6$ vs $11.0 \pm 5.0 \times 10^{-6}$ fluorescence A.U per gram tumour) (Duska et al, 1997). However, due to the chemical modification, the tumour uptake of the conjugate was lower than that of the native F(ab')$_2$ fragments. Twenty four hours post injection, the %ID/g tumour was $38.1 \pm 2.9$, $17.1 \pm 1.8$ and $3.8 \pm 0.9$ for the OC125 F(ab')$_2$, the cationic conjugate and the anionic conjugate, respectively. Recently, these authors moved to human colon carcinoma as a target tumour. Using MAb 17.1A, they confirmed the higher in vitro photo-induced cytotoxicity of a cationic conjugate as compared to an anionic conjugate on HT29 cells (Del Governatore, 2000b). In vivo, using hepatic metastases induced with HT29 cells in nude mice, the anionic conjugate delivered 5 and 8 times more $c_{e6}$ than the cationic conjugate at 3 and 24 h post-i.v. injection, respectively (Hamblin et al, 2000). The discrepancy between the in vitro and the in vivo results in this model was attributed to the use of the i.v. route of injection of the conjugate which induced a high $c_{e6}$ uptake in lung and bladder. Therefore, the authors used the anionic conjugate in an immunophototherapy study in which the anionic 17.1A-$c_{e6}$ was shown to be more efficient than free $c_{e6}$ to enhance the median survival time of nude mice bearing HT29 induced liver metastases (62.5, 77 and 102 days for the control group, the $c_{e6}$ treated group and the anionic 17.1A-$c_{e6}$ conjugate treated group, respectively) (Del Governatore, 2000a). The overall results of these studies underlined (i) the potential of chemically modifying MAb with conjugated dyes to enhance their photo-induced cytotoxicity; (ii) the technical difficulties inherent to this approach. The cationization of MAb-dye conjugates is a chemical modification that is not related to the antigen specificity of the MAb. It can hence increase internalisation of the conjugate in different cell types.

Another approach to deliver phototoxic dyes into cells is the use of MAb directed against internalising antigens. This strategy was

 © 2001 Cancer Research Campaign

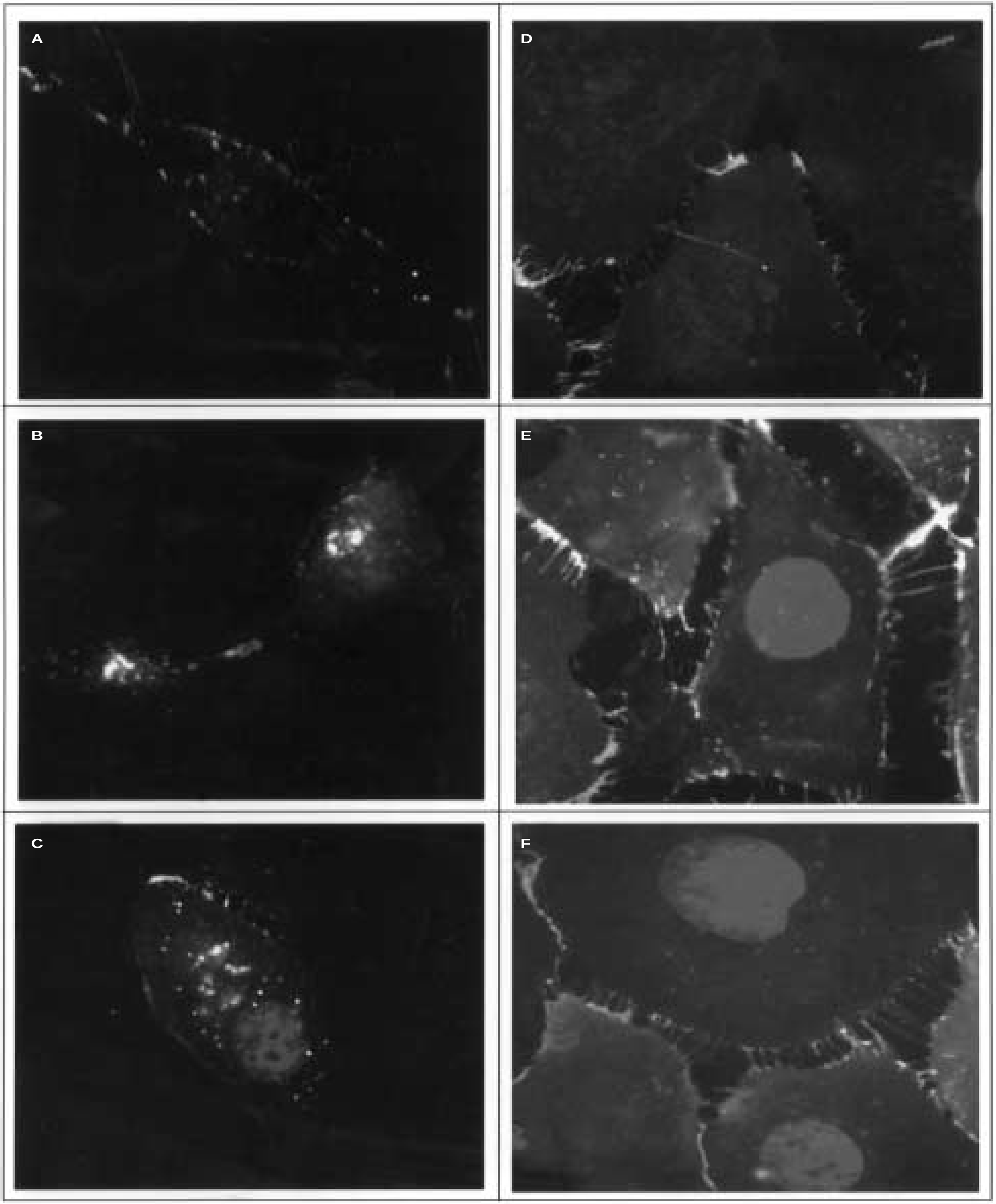

**Figure 2** Photomicrograph of SKOv3-CEA-1B9 cell line. Cells were grown on coverslips, fixed and stained with anti-mouse Ig FITC conjugated-green fluorescence and DAPI-blue nuclear stained. Uptake of the FSP77-$(AlPcS_4A_1)_6$ conjugate is shown after incubation at 37˚C for 30 min (**A**), 90 min (**B**) and 240 min (**C**). Uptake of the 35A7-$(AlPcS_4A_1)_8$ conjugate is shown after at 37˚C at 30 min (**D**), 90 min (**E**) and 240 min (**F**)

first addressed by Vrouenraets et al with *m*THPC as photosensitizer suggesting that in vitro a conjugate prepared with the internalising MAb 425 (anti-EGF-R) was more phototoxic than a non-internalising MAb prepared with MAb U36 directed against the v6 domain of the 200 kDa CD44 splice variant epican (Vrouenraets et al, 1999). However these results, which were

© 2001 Cancer Research Campaign

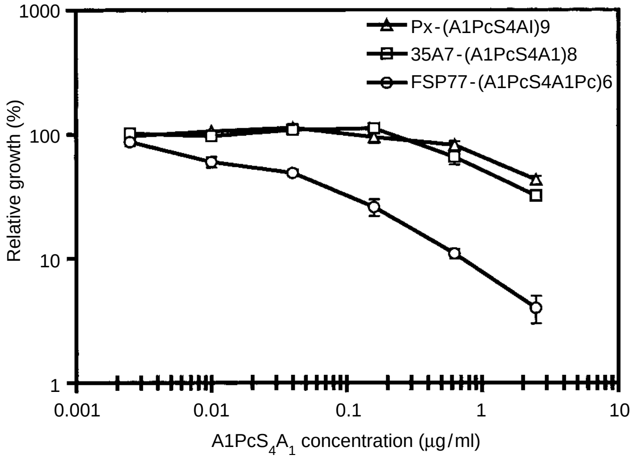

**Figure 3** Photo-induced cytotoxicity of FSP77-($AlPcS_4A_1$)$_6$ (○), 35A7-($AlPcS_4A_1$)$_8$ (❑) and Px-($AlPcS_4A_1$)$_9$ (Δ). The SKOv3-CEA-1B9 cells were incubated with immunoconjugates at 37˚C for 20 h at six different concentrations of $AlPcS_4A_1$ ranging from 0.0025 to 2.5 µg/ml. After two washes with cold phenol red-free RPMI, the cells were kept at 37˚C and illuminated with 50 J/cm$^2$. Cell survival was estimated by the colorimetric MTT assay after 48 h at 37˚C

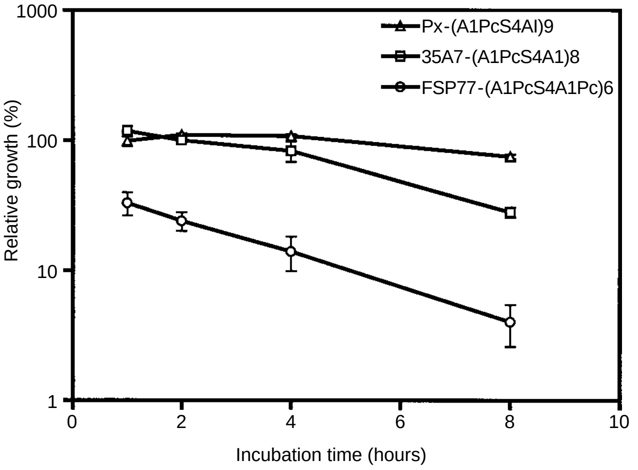

**Figure 4** Photo-induced cytotoxicity of FSP77-($AlPcS_4A_1$)$_6$ (○), 35A7-($AlPcS_4A_1$)$_8$ (□) and Px-($AlPcS_4A_1$)$_9$ (△). The SKOv3-CEA-1B9 cells were incubated with immunoconjugates at 37˚C for 1, 2, 4 and 8 h with a constant concentration of $AlPcS_4A_1$ of 1.25 µg/ml. After two washings with cold phenol red-free RPMI, the cells were kept at 37˚C and illuminated with 50 J/cm$^2$. Cell survival was estimated by the colorimetric MTT assay after 48 h at 37˚C

obtained on two different cell lines (A431 for MAb 425 and UM-SCC-22A for U36), did not take into account the specific photosensitivity of these two cell lines. Furthermore, free *m*THPC was as efficient as MAb 425-*m*THPC conjugate in killing A431 cells. In a second study, the authors used an hydrophilic porphyrin derivative, TrisMPyP-Φ$CO_4$H, to demonstrate in vitro that internalising conjugates prepared with MAb 425 and MAb U36 exhibited a higher photo-induced cytotoxicity on A431 cells than the free dye and a non-internalising conjugate prepared with MAb E48 directed against a surface antigen expressed by HNSCC (Vrouenraets et al, 2000). The antigenic density of these different target antigens on A431 cells as well as the affinity constants of the different MAbs against their antigen were not clearly mentioned in the manuscript but these data confirmed the potential advantage of internalising over non-internalising conjugates. The major limitation of the TrisMPyP-Φ$CO_2$H-MAb conjugates is their poor tumour uptake as compared to the native MAb. In HNX-OE SCC bearing nude mice, MAb U36 and conjugates loaded with 1.2, 2.1 and 3.0 TrisMPyP-Φ$CO_2$H per MAb showed tumour uptake values of 15.5, 8.6, 6.5 and 4.0%ID/g, respectively.

In the present study, using our $AlPcS_4A_1$ hydrophilic photosensitizer, we compared the in vitro photo-induced cytotoxicity of an internalising (MAb FSP77) to that of a non-internalising conjugate (MAb 35A7) on the same cell line SKOv3-CEA-1B9. This cell line, which was derived from the human ovarian cancer SKOv3 by transfection with the CEA cDNA, was shown to express the two target antigens, ErbB2 and CEA, at the same high level (Figure 1). As demonstrated by immunofluorescence, the FSP77-($AlPcS_4A_1$)$_6$ conjugate internalised rapidly in SKOv3-CEA-1B9 cells whereas the 35A7-($AlPcS_4A_1$)$_8$ conjugate did not (Figure 2). The non-internalising conjugate was phototoxic but the higher photo-induced cytotoxicity of the internalising conjugate was demonstrated in a dose dependent experiment (Figure 3) as well as a time dependent study (Figure 4). These data confirm the advantage of anti-Erb2 FSP77-$AlPcS_4A_1$ conjugates over anti-CEA 35A7-$AlPcS_4A_1$ conjugates for photodynamic applications. The major advantage of our photosensitizer is that conjugates bearing up to 16 $AlPcS_4A_1$ per MAb were shown to give tumour uptake values similar to that obtained with the native MAb (Carcenac et al, 1999). On the basis of these in vitro results, anti-ErbB2-$AlPcS_4A_1$ conjugates are currently evaluated for their therapeutic potential on breast and ovary carcinoma.

### ACKNOWLEDGEMENTS

We thank Mrs Sabine Bousquié, Mr Michel Brissac, Mrs Geneviève Heintz and Mrs Céline Passet for their expert technical assistance, Dr Christophe Duperray for cell sorting. Generous financial support by the Caisse d'Assurances Maladie des Professions Libérales Provinces (CAMPLP), the Ligue Nationale Contre le Cancer (Comité de l'Hérault) and the Canadian Institutes of Health Research is gratefully acknowledged.

### REFERENCES

Brasseur N, Langlois R, La Madeleine C, Ouellet R and van Lier JE (1999) Receptor-mediated targeting of phthalocyanines to macrophages via covalent coupling to native or maleylated bovine serum albumin. *Photochem Photobiol* **69**: 345–352

Carcenac M, Larroque C, Langlois R, Van Lier JE, Artus JC and Pèlegrin A (1999) Preparation, phototoxicity and biodistribution studies of anti-CEA MAb-phthalocyanine conjugates. *Photochem Photobiol* **70**: 930–936

Del Governatore M, Hamblin MR, Shea CR, Rizvi I, Molpus KG, Tanabe KK and Hasan T (2000a) Experimental photoimmunotherapy of hepatic metastases of colorectal cancer with a 17.1A chlorin e6 immunoconjugate. *Cancer Res* **60**: 4200–4205

Del Governatore M, Hamblin MR, Piccinini EE, Ugolini G and Hasan T (2000b) Targeted photodestruction of human colon cancer cells using charged 17.1A chlorin e6 immunoconjugates. *Br J Cancer* **82**: 56–64

Dougherty TJ, Gomer CJ, Henderson BW, Jori G, Kessel D, Korbelik M, Moan J and Peng Q (1998) Photodynamic therapy. *J Natl Cancer Inst* **90**: 889–905

Duska LR, Hamblin MR, Bamberg MP and Hasan T (1997) Biodistribution of charged F(ab')2 photoimmunoconjugates in a xenograft model of ovarian cancer. *Br J Cancer* **75**: 837–844

Goff BA, Hermanto U, Rumbaugh J, Blake J, Bamberg M and Hasan T (1994) Photoimmunotherapy and biodistribution with an OC125-chlorin immunoconjugate in an in vivo murine ovarian cancer model. *Br J Cancer* **70**: 474–480

Gutowski M, Carcenac M, Pourquier D, Larroque C, Rouanet P and Pèlegrin A (2001) Intraoperative immunophotodetection for radical resection of cancer: evaluation in an experimental model. *Clin Cancer Res* In Press

*© 2001 Cancer Research Campaign*

Hamblin MR, Del Governatore M, Rizvi I and Hasan T (2000) Biodistribution of charged 17.1A photoimmunoconjugates in a murine model of hepatic metastasis of colorectal cancer. *Br J Cancer* **83**: 1544–1551

Hamblin MR, Miller JL and Hasan T (1996) Effect of charge on the interaction of site-specific photoimmunoconjugates with human ovarian cancer cells. *Cancer Res* **56**: 5205–5210

Harwerth IM, Wels W, Marte BM and Hynes NE (1992) Monoclonal antibodies against the extracellular domain of the erbB-2 receptor function as partial ligand agonists. *J Biol Chem* **267**: 15160–15167

Kohler G, Howe SC and Milstein C (1976) Fusion between immunoglobulin-secreting and nonsecreting myeloma cell lines. *Eur J Immunol* **6**: 292–295

Merlin JL, Azzi S, Lignon D, Ramacci C, Zeghari N and Guillemin F (1992) MTT assays allow quick and reliable measurement of the response of human tumour cells to photodynamic therapy. *Eur J Cancer* **28A**: 1452–1458

Mew D, Wat CK, Towers GHN and Levy JG (1983) Photoimmunotherapy: treatment of animal tumors with tumor-specific monoclonal antibody-hematoporphyrin conjugates. *J Immunol* **130**: 1473–1477

Oseroff AR, Ohuoha D, Hasan T, Bommer JC and Yarmush ML (1986) Antibody-targeted photolysis: selective photodestruction of human T-cell leukemia cells using monoclonal antibody-chlorin e6 conjugates. *Proc Natl Acad Sci USA* **83**: 8744–8748

Pèlegrin A, Folli S, Buchegger F, Mach JP, Wagnières G and van den Bergh H (1991) Antibody-fluorescein conjugates for photoimmunodiagnosis of human colon carcinoma in nude mice. *Cancer* **67**: 2529–2537

Pèlegrin A, Terskikh A, Hayoz D, Chalandon Y, Olsson NO, Folli S, Buchegger F, Kromer B, Schwarz K, Martin M, Martin F and Mach JP (1992) Human carcinoembryonic antigen cDNA expressed in rat carcinoma cells can function as target antigen for tumor localization of antibodies in nude rats and as rejection antigen in syngeneic rats. *Int J Cancer* **52**: 110–119

Vrouenraets MB, Visser GW, Loup C, Meunier B, Stigter M, Oppelaar H, Stewart FA, Snow GB and van Dongen GA (2000) Targeting of a hydrophilic photosensitizer by use of internalizing monoclonal antibodies: A new possibility for use in photodynamic therapy. *Int J Cancer* **88**: 108–114

Vrouenraets MB, Visser GW, Stewart FA, Stigter M, Oppelaar H, Postmus PE, Snow GB and van Dongen GA (1999) Development of meta-tetrahydroxy-phenylchlorin-monoclonal antibody conjugates for photoimmunotherapy. *Cancer Res* **59**: 1505–1513

© 2001 Cancer Research Campaign
